# JAK2 Inhibition by Fedratinib Reduces Osteoblast Differentiation and Mineralisation of Human Mesenchymal Stem Cells

**DOI:** 10.3390/molecules26030606

**Published:** 2021-01-25

**Authors:** Nihal AlMuraikhi, Hanouf Alaskar, Sarah Binhamdan, Amal Alotaibi, Moustapha Kassem, Musaad Alfayez

**Affiliations:** 1 Stem Cell Unit, Department of Anatomy, College of Medicine, King Saud University, Riyadh 11461, Saudi Arabia; 437203446@student.ksu.edu.sa (H.A.); sarahbinhamdan@gmail.com (S.B.); amal.otb.1432@gmail.com (A.A.); mkassem@health.sdu.dk (M.K.); alfayez@ksu.edu.sa (M.A.); 2 College of Science, King Saud University, Riyadh 11461, Saudi Arabia; 3 College of Medicine, Alfaisal University, Riyadh 11533, Saudi Arabia; 4 Molecular Endocrinology Unit (KMEB), Department of Endocrinology, University Hospital of Odense, University of Southern Denmark, 5000 Odense C, Denmark; 5 Department of Cellular and Molecular Medicine, Danish Stem Cell Center (DanStem), University of Copenhagen, 2200 Copenhagen, Denmark

**Keywords:** JAK/STAT signalling pathway, JAK2 inhibition, Fedratinib, hMSC-TERT, osteoblast differentiation, in vitro mineralisation

## Abstract

Several signalling pathways, including the JAK/STAT signalling pathway, have been identified to regulate the differentiation of human bone marrow skeletal (mesenchymal) stem cells (hBMSCs) into bone-forming osteoblasts. Members of the JAK family mediate the intracellular signalling of various of cytokines and growth factors, leading to the regulation of cell proliferation and differentiation into bone-forming osteoblastic cells. Inhibition of JAK2 leads to decoupling of its downstream mediator, STAT3, and the subsequent inhibition of JAK/STAT signalling. However, the crucial role of JAK2 in hBMSCs biology has not been studied in detail. A JAK2 inhibitor, Fedratinib, was identified during a chemical biology screen of a small molecule library for effects on the osteoblastic differentiation of hMSC-TERT cells. Alkaline phosphatase activity and staining assays were conducted as indicators of osteoblastic differentiation, while Alizarin red staining was used as an indicator of in vitro mineralised matrix formation. Changes in gene expression were assessed using quantitative real-time polymerase chain reaction. Fedratinib exerted significant inhibitory effects on the osteoblastic differentiation of hMSC-TERT cells, as demonstrated by reduced ALP activity, in vitro mineralised matrix formation and downregulation of osteoblast-related gene expression, including ALP, ON, OC, RUNX2, OPN, and COL1A1. To identify the underlying molecular mechanisms, we examined the effects of Fedratinib on a molecular signature of several target genes known to affect hMSC-TERT differentiation into osteoblasts. Fedratinib inhibited the expression of LIF, SOCS3, RRAD, NOTCH3, TNF, COMP, THBS2, and IL6, which are associated with various signalling pathways, including TGFβ signalling, insulin signalling, focal adhesion, Notch Signalling, IL-6 signalling, endochondral ossification, TNF-α, and cytokines and inflammatory response. We identified a JAK2 inhibitor (Fedratinib) as a powerful inhibitor of the osteoblastic differentiation of hMSC-TERT cells, which may be useful as a therapeutic option for treating conditions associated with ectopic bone formation or osteosclerotic metastases.

## 1. Introduction

Janus kinase (JAK)/signalling transducers and activators of transcription (STAT) signalling has been identified as the downstream signalling of a wide array of growth factors, hormones, and cytokines (such as interferon-γ and interleukin-6 family members) that regulate different cellular processes, including cell proliferation, differentiation, cellular senescence, and apoptosis [1,2,3,4,5,6,7,8]. The JAK family is composed of four members, JAK1, JAK2, JAK3 and the tyrosine-protein kinase 2 (Tyk2). These proteins are widely expressed, with the exception of JAK3, which is highly expressed in leukocytes and haematopoietic cells [1,3,4,6,7]. The members of this family have a unique structure of four domains, i.e., the FERM, SH2, pseudokinase, and kinase domains [4,8,9]. In addition, they have two adjacent kinase domains, JH1 and JH2; JH1 is responsible for the phosphorylation required for pathway activation, while JH2 controls JH1 function [4]. In humans, the STAT family has seven members, STAT1, STAT2, STAT3, STAT4, STAT5α, STAT5β, and STAT6, that contain SH2 functional domains to enable binding to tyrosine residues and C-terminal domains and activate transcription [1,3,4,8]. Thus, binding of cytokines and growth factors to their cognate cell-surface receptors initiates the JAK/STAT signalling pathway through the phosphorylation of STAT, as the intracellular domains of the cytokine receptors are linked to tyrosine kinases of the JAK family [3,4,7]. For example, receptors of the interleukin-6 family cytokines contain a transmembrane protein subunit, gp130, that is activated upon receptor binding and triggers the activation of gp130-associated JAK1, JAK2, and TYK2 [3,7,10].

hBMSCs are multipotent stem cells that reside in the bone marrow and are capable to differentiate into mesodermal cells, including bone-forming osteoblasts [11,12]. Various signalling pathways have been found to regulate the osteoblastic differentiation of hBMSCs [13,14] including JAK/STAT signalling [15], Wnt/β-catenin [16], TGFβ [17], insulin signalling [18], focal adhesion [19], Notch Signalling [20], IL-6 signalling [21], and Hh signalling [22]. 

JAK/STAT signalling plays an important role in bone development. It has been shown that inhibition of JAK2 disconnects it from the downstream mediator STAT3, leading to the inhibition of hMSCs differentiation into osteoblasts [8,23]. 

Small molecule inhibitors targeting specific intracellular signalling pathways are currently used in vitro as a biochemical tool to identify the molecular mechanisms involved in stem cell differentiation into osteoblast lineages [15,20,22,24,25,26,27]. Here, we identified a small molecule Fedratinib, a potent JAK2 inhibitor [28], through a small molecule library screen [15], as a powerful inhibitor of osteoblastic differentiation of hMSC-TERT cells.

## 2. Results

### 2.1. Effect of Fedratinib on hMSC-TERT Cell Proliferation

**Figure 1:**
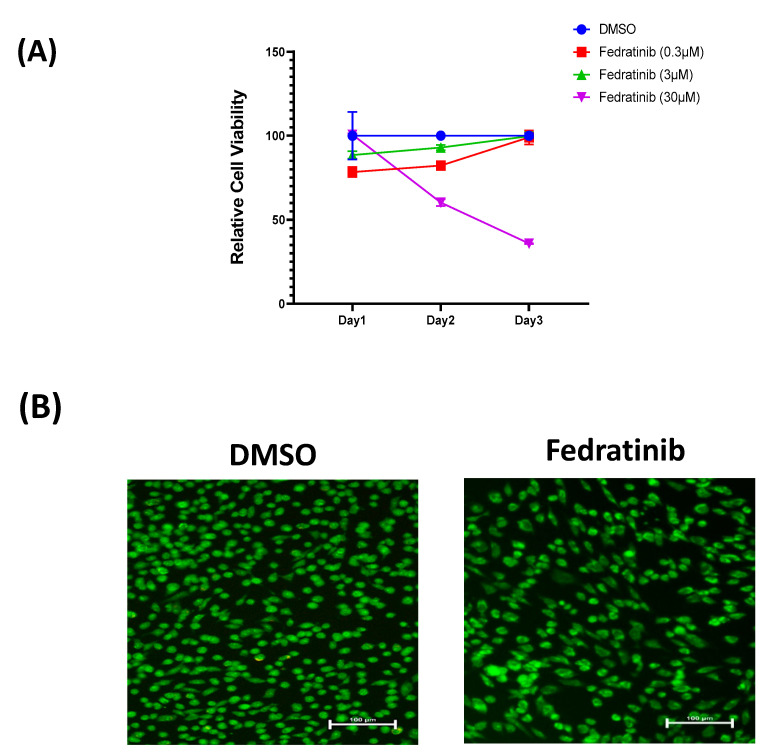
Effects of Fedratinib treatment on the number of hMSC-TERT cells. (**A**) Dose-response curve of hMSC-TERT cells to different doses of Fedratinib, as indicated in the graph, vs. DMSO-treated control cells as measured by cell number assay over 3 days. (**B**) Representative fluorescence images of Fedratinib-treated hMSC-TERT cells (3.0 µM) vs. DMSO-treated control cells on day 3 of exposure. Photomicrograph magnification, 20×. Cells were stained with AO/EtBr to detect apoptotic (cells with green condensed chromatin) and necrotic (red) cells. DMSO: dimethyl sulfoxide.

Previously, we reported the results of a small molecule library screen that recognised various small molecule inhibitors with diverse effects on the osteoblastic differentiation of hMSC-TERT cells using Alkaline phosphatase (ALP) activity quantification as a read-out [15]. Among them, Fedratinib, a JAK2 inhibitor, exhibited potent inhibitory effects. Thus, we studied Fedratinib comprehensively. We first studied the effect of continuous exposure to in vitro treatment of hMSC-TERT with Fedratinib at increasing concentrations (0.3–30 µM) for 1, 2, and 3 days, on hMSC-TERT cell viability (Figure 1A). The cell viability assay used here reflects cell number, which is a good indicator of cell proliferation. We observed no major effects of Fedratinib on hMSC-TERT cell viability at doses of 0.3 and 3 μM. However, Fedratinib decreased hMSC-TERT cell viability at a dose of 30 μM after 3 days of treatment. To determine the underlying cellular mechanism, we conducted an apoptosis assay after Fedratinib treatment (3 µM) for 3 days, but did not observe significant changes in the number of apoptotic cells compared with DMSO-vehicle treated control cells and this concentration did not cause cell death (Figure 1B).

### 2.2. Fedratinib Inhibits the Osteoblastic Differentiation of hMSC-TERT Cells

**Figure 2:**
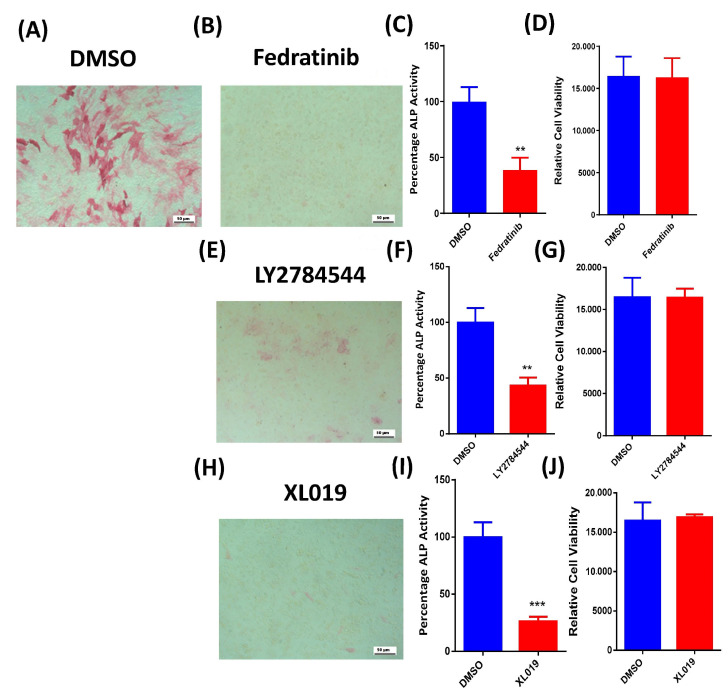
Effects of JAK2 inhibitors on the osteoblastic differentiation of hMSC-TERT cells. (**A**) Representative ALP staining of DMSO-treated control cells on day 10 post-osteoblastic differentiation. Photomicrograph magnification, 10×. (**B**) Representative ALP staining of Fedratinib-treated hMSC-TERT cells (3.0 µM) on day 10 post-osteoblastic differentiation. Photomicrograph magnification, 10×. (**C**) Quantification of ALP activity in Fedratinib-treated hMSC-TERT cells (3.0 µM) vs. DMSO-treated control cells on day 10 post-osteoblastic differentiation. Data are presented as the mean percentage of ALP activity ± SEM (n = 20). (**D**) Assay of cell viability using alamarBlue in Fedratinib-treated hMSC-TERT cells (3.0 µM) vs. DMSO-treated control cells on day 10 post-osteoblastic differentiation. Data are presented as the mean ± SEM (n = 20). (**E**) Representative ALP staining of LY2784544-treated hMSC-TERT cells (3.0 µM) on day 10 post-osteoblastic differentiation. Photomicrograph magnification, 10×. (**F**) Quantification of ALP activity in LY2784544-treated hMSC-TERT cells (3.0 µM) vs. DMSO-treated control cells on day 10 post-osteoblastic differentiation. Data are presented as the mean percentage of ALP activity ± SEM (n = 20). (**G**) Assay of cell viability using alamarBlue assay in LY2784544-treated hMSC-TERT cells (3.0 µM) vs. DMSO-treated control cells on day 10 post-osteoblastic differentiation. Data are presented as the mean ± SEM (n = 20). (**H**) Representative ALP staining of XL019-treated hMSC-TERT cells (3.0 µM) on day 10 post-osteoblastic differentiation. Photomicrograph magnification, 10×. (**I**) Quantification of ALP activity in XL019-treated hMSC-TERT cells (3.0 µM) vs. DMSO-treated control cells on day 10 post-osteoblastic differentiation. Data are presented as the mean percentage ALP activity ± SEM (n = 20). (**J**) Assay of cell viability using alamarBlue assay in XL019-treated hMSC-TERT cells (3.0 µM) vs. DMSO-treated control cells on day 10 post-osteoblastic differentiation. Data are presented as the mean ± SEM (n = 20). ALP, alkaline phosphatase; DMSO, dimethyl sulfoxide. ** *p* < 0.005; *** *p* < 0.0005.

**Figure 3:**
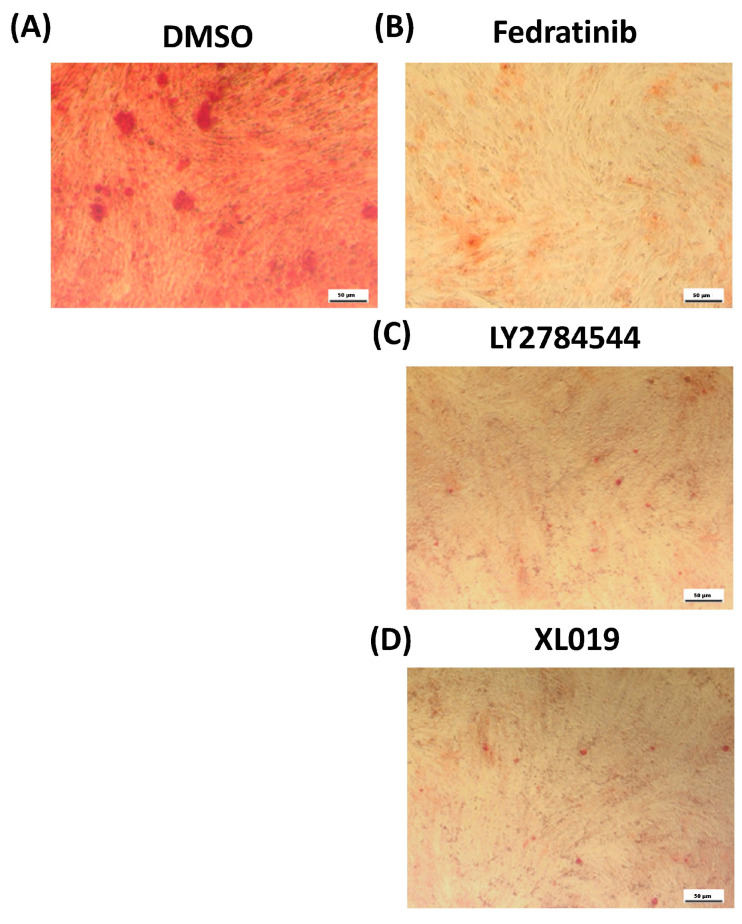
Effects of JAK2 inhibitors on the mineralisation of hMSC-TERT cells. (**A**) Cytochemical staining of mineralised matrix formation using Alizarin red on day 21 post-osteoblastic differentiaTable 21. post-osteoblastic differentiation in Fedratinib-treated hMSC-TERT cells (3.0 µM). (**C**) Cytochemical staining of mineralised matrix formation using Alizarin red on day 21 post-osteoblastic differentiation in LY2784544-treated hMSC-TERT cells (3.0 µM). (**D**) Cytochemical staining of mineralised matrix formation using Alizarin red on day 21 post-osteoblastic differentiation in XL019-treated hMSC-TERT cells (3.0 µM). Photomicrograph magnification, 10×.

**Figure 4:**
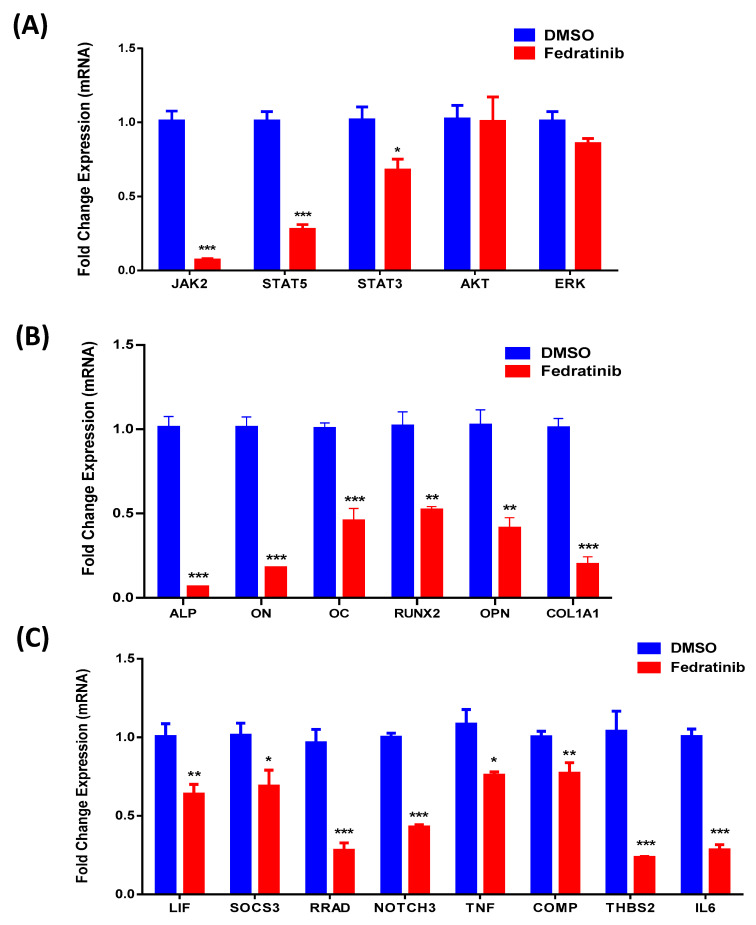
Fedratinib downregulates multiple osteoblast-associated genes. (**A**) Quantitative RT-PCR analysis of the expression of JAK2, STAT5, STAT3, AKT, and ERK in hMSC-TERT cells after 24 h of osteoblastic differentiation in the absence (blue) or presence (red) of Fedratinib (3.0 µM). (**B**) Quantitative RT-PCR analysis of the expression of ALP, ON, OC, RUNX2, OPN, and COL141 in hMSC-TERT cells on day 10 post osteoblastic differentiation in the absence (blue) or presence (red) of Fedratinib (3.0 µM). Alkaline phosphatase (ALP); Osteonectin (ON); Osteocalcin (OC); Runt-related transcription factor 2 (RUNX2); Osteopontin (OPN); Collagen type I alpha 1 (COL1A1); Dimethyl sulfoxide (DMSO). (**C**) Quantitative RT-PCR analysis of a selected panel of osteoblast differentiation-associated genes in Fedratinib-treated hMSC-TERT cells vs. DMSO-treated control cells using qRT-PCR on day 10 post osteoblastic differentiation in the absence (blue) or presence (red) of Fedratinib (3.0 µM). Gene expression was normalised to that of β-actin. Data are presented as the mean fold change ± SEM (n = 6) in replicates from two independent experiments; * *p* < 0.05; ** *p* < 0.005; *** *p* < 0.0005.

Fedratinib-treated hMSC-TERT cells (3 µM) exhibited a significant reduction in ALP production, as evidenced by reduced cytochemical staining intensity (Figure 2B) compared with DMSO-vehicle treated control cells (Figure 2A). Moreover, the measurement of ALP activity at day 10 post-osteoblast differentiation induction was reduced compared with DMSO-vehicle treated control cells (Figure 2C). Fedratinib did not exert significant effects on hMSC-TERT cells viability at day 10 post-osteoblast differentiation induction (Figure 2D). Fedratinib-treated hMSC-TERT cells (3 µM) exhibited a significant decrease in mineralised matrix formation, as demonstrated by Alizarin red staining (Figure 3B), compared with DMSO-vehicle treated control cells (Figure 3A), which was associated with the significant downregulation of several osteoblast gene markers: ALP, ON, OC, RUNX2, OPN, and COL1A1 (Figure 4B).

To confirm our findings, we tested the effect of an additional two JAK2 inhibitors, LY2784544 and XL019, on the osteoblastic differentiation of hMSC-TERT cells. Both treatments yielded significant reductions in ALP production as evidenced by reduced cytochemical staining intensity (Figure 2E,H) compared with DMSO-vehicle treated control cells (Figure 2A). Moreover, ALP activity measurement at day 10 post-osteoblast differentiation induction was also reduced compared with DMSO-vehicle treated control cells (Figure 2F,I). However, no significant effects on hMSC-TERT cell viability were measured at day 10 post-osteoblast differentiation induction (Figure 2G,J). Moreover, hMSC-TERT cells treated with LY2784544 and XL019 exhibited a significant decrease in mineralised matrix formation as demonstrated by Alizarin red staining (Figure 3C,D) compared with DMSO-vehicle-treated control cells (Figure 3A).

### 2.3. Fedratinib Affects Multiple Signalling Pathways During the Osteoblastic Differentiation of hMSC-TERT Cells

To confirm that Fedratinib targeted the JAK2 signalling pathway, hMSC-TERT cells were treated with Fedratinib at the same concentration (3 μM), and 24 h later, the expression of JAK2, STAT5, STAT3, AKT, and ERK, which are downstream readouts of the JAK2 signalling pathway, was assessed using qRT-PCR. The data presented in Figure 4A demonstrated significant inhibition of the JAK2 signalling pathway by Fedratinib, as evidenced by the suppression of JAK2, STAT5 and STAT3 expression. These data suggest that Fedratinib exerts inhibitory effects on osteoblast differentiation and mature osteoblastic cell functions through the inhibition of the JAK2 signalling pathway.

We have previously reported several small molecule inhibitors with inhibitory effects on the osteoblastic differentiation of hMSC-TERT cells as shown by global gene expression profiling [15,20,22]. Various significantly enriched signalling pathways that are known to regulate the osteoblastic differentiation of hMSC-TERT cells were identified including TGFβ signalling, insulin signalling, focal adhesion, Notch Signalling, IL-6 signalling, endochondral ossification, TNF-α, and cytokines and inflammatory response. Thus, we sought to evaluate the effect of Fedratinib on these signalling pathways by examining the expression of a gene signature (Figure 4C). LIF, SOCS3, RRAD, NOTCH3, TNF, COMP, THBS2, and IL6 were significantly downregulated in Fedratinib-treated hMSC-TERT cells (3 µM) compared with DMSO vehicle-treated control cells on day 10 of osteoblastic differentiation.

## 3. Discussion

hBMSCs are multipotent cells that can differentiate into bone-forming osteoblastic cells during bone formation and remodelling [11,12]. Understanding the molecular signalling pathways underlying osteoblastic differentiation is relevant for determining the pathogenesis of skeletal diseases and to discover novel therapies [13,14]. Small molecule inhibitors targeting specific intracellular signalling pathways may be employed to identify key molecular targets and as part of cellular differentiation protocols because of their low cost, stability, and ease of use [15]. In the present study, Fedratinib, which was discovered during a small molecule library functional screen, was found to be a strong inhibitor of hMSC-TERT cells differentiation to osteoblastic cells.

Fedratinib is an oral JAK2 inhibitor [28] that has been recently approved by the Food and Drug Administration to treat adults with myelofibrosis based on the significant improvements reported in phase II and III clinical trials, which renders this drug the second to be approved for myelofibrosis, after ruxolitinib, a JAK1/2 inhibitor. Fedratinib exhibited clinical efficiency in ruxolitinib-resistant patients [29,30]. Myelofibrosis is a myeloproliferative neoplasm characterised by bone-marrow fibrosis, cytopenia, extramedullary hematopoiesis, and splenomegaly [29]. The main side effects associated with Fedratinib treatment are anaemia, gastrointestinal complications, high liver transaminases, and occasionally encephalopathy [29]. However, no bone-related complications have been reported to date. 

We observed that Fedratinib treatment reduced osteoblastic differentiation, in vitro mineralisation, and the expression of osteoblast-related genes. The role of the JAK/STAT signalling pathway in triggering osteoblastic differentiation has been widely described [1,2,3,5,15,23,31,32]. 

Osteoblastic differentiation, bone development, mineralisation, and fracture repair can be mediated by several intracellular signalling pathways, such as the JAK/STAT pathway [23,31]. The in vitro activation of the JAK/STAT signalling can promote ALP activity, and increase OC expression, suggesting a vital role for JAK/STAT in osteoblastic differentiation [23]. Moreover, the fracture-healing capacity was impaired in JAK2-deficient mice. A plausible explanation for this observation is that inhibiting the expression of JAK2 leads to decoupling from STAT3, consequently inhibiting the osteoblastic differentiation of hBMSCs [23,33]. In addition, the inhibition of JAK2 after the in vitro osteogenic differentiation of hBMSCs inhibited the production of osteoblasts, demonstrating the importance of JAK2/STAT3 signalling in the osteoblastic differentiation of hBMSCs [23]. 

JAK2 phosphorylation stimulates several signalling pathways, including STAT3, STAT5, the phosphoinositide-3 kinase (PI3K)/AKT pathway and the mitogen activated protein kinase pathway involving ERK kinases, which control cell survival, proliferation and differentiation [34]. STAT5 and STAT3 are downstream effectors of various tyrosine kinase oncogenes including JAK2 [35,36]. Thus, they are robust pharmacodynamic biomarkers of baseline JAK2 activity and of the efficacy of chemical JAK2 inhibition [37]. Inhibiting JAK2 reduces cell proliferation and induces apoptosis in cancer cells because of inhibition of the phosphorylation of the JAK2 substrates STAT3 and STAT5 [38]. Fedratinib reduced the phosphorylation of downstream STAT3/5 proteins in cell models [39,40]. The JAK2 pathway can directly or indirectly affect various osteoblast differentiation mediators and markers, including BMPs, ALP, Runx2, and OPN [1]. In addition, loss of JAK2 activation can be correlated with the loss of activation of other downstream signalling mediators such as AKT and ERK. JAK2 inhibition suppresses MEK/ERK activation in vitro [1,34,39,41]. 

Global gene expression profiling of hMSC-TERT cells after treatment with previously reported small molecules that inhibit osteoblast differentiation [15,20,22] identified significant changes in multiple osteoblast differentiation-related intracellular signalling pathways including TGF-β signalling [17], insulin signalling [18], focal adhesion [19], Notch signalling [20], IL-6 signalling [21], endochondral ossification [42], TNF-α [43], and cytokines and inflammatory response [44]. Therefore, in the present study, we sought to evaluate the effect of Fedratinib on the reported signalling pathways by examining a molecular gene signature, i.e., significant downregulation of LIF, SOCS3, RRAD, NOTCH3, TNF, COMP, THBS2, and IL-6, suggesting that treatment with Fedratinib leads to changes in the expression of several genes associated with multiple signalling pathways. While it is plausible that these changes are secondary to effects on JAK2 signalling, additional experiments need to be performed to support this hypothesis.

## 4. Materials and Methods 

### 4.1. Cell Culture

We used the hMSC-TERT cell line as a model of hBMSCs in all experiments included in this study. The hMSC-TERT cell line was generated by overexpressing the human telomerase reverse transcriptase gene (hTERT). The hMSC-TERT cell line retains all the distinctive features of primary hBMSCs, including infinite self-renewal and potency, and expresses all gene markers [45,46].

Cells were maintained in Dulbecco’s Modified Eagle Medium (DMEM), a basal medium supplemented with 4500 mg/L *D*-glucose, 110 mg/L 10% sodium pyruvate, 4 mM *L*-glutamine, 10% fetal bovine serum (FBS), 1% non-essential amino acids, and 1% penicillin-streptomycin, as previously defined [46]. All reagents were obtained from Thermo Fisher Scientific Life Sciences, Waltham, MA. Cells were incubated in 5% CO_2_ incubators at 37 °C and 95% humidity. 

### 4.2. Osteoblastic Differentiation

Cells were maintained to reach 85% confluence before replacing the medium with osteoblast induction medium containing DMEM supplemented with 50 mg/mL *L*-ascorbic acid (Wako Chemicals GmbH, Neuss, Germany), 10 mM *b*-glycerophosphate (Sigma-Aldrich, St. Louis, MO, USA), 10 nM calcitriol (1a,25-dihydroxyvitamin D_3_; Sigma-Aldrich), and 10 nM dexamethasone (Sigma-Aldrich), in addition to 10% FBS, and 1% penicillin-streptomycin, as previously defined [15]. The stem cell signalling library including the Fedratinib, LY2784544, and XL019 small molecule inhibitors was obtained from Selleckchem Inc. Small molecule inhibitors were added separately at a concentration of 3 µM to the osteoblast induction medium and cells were constantly exposed to the inhibitor during the differentiation period. Control cells were maintained in osteoblast induction medium containing dimethyl sulfoxide (DMSO) as a vehicle.

### 4.3. Cell Viability Assay

Cell viability analysis was conducted using the alamarBlue method according to the manufacturer’s recommendations (Thermo Fisher Scientific Life Sciences, Waltham, MA, USA) as previously defined [15]. To obtain a dose-response growth curve, cells were maintained in 96-well plates in 300 μL of the medium containing different concentrations of Fedratinib (0.3, 3, and 30 μM) compared with DMSO-vehicle-treated control cells. At different time points (days 1, 2, and 3), 30 μL/well of the alamarBlue substrate was added (10%) and cells were incubated for 1 h at 37 °C in the dark before readings using a BioTek Synergy II microplate reader (BioTek Inc., Winooski, VT, USA) in the fluorescent mode (Ex 530 nm/Em 590 nm). For cell viability, cells were maintained in 96-well plates in 300 μL of the medium. On day 10, 30 μL/well of the alamarBlue substrate was added (10%) and cells were incubated for 1 h at 37 °C in the dark before readings using a BioTek Synergy II microplate reader (BioTek Inc., Winooski, VT, USA) in the fluorescent mode (Ex 530 nm/Em 590 nm).

### 4.4. Measurement of Apoptosis

Cell apoptosis was measured by a fluorescence-based assay using the acridine orange/ethidium bromide (AO/EtBr) dyes as previously defined [47], in cells treated with Fedratinib (3 µM) compared with DMSO-vehicle-treated control cells. On day 3 post treatment, cells were mixed with a dual fluorescent dye (1.0 µL) containing 100 µg/mL of AO and 100 µg/mL of EtBr (AO/EB, Sigma, St. Louis, MO, USA) for 1 min before they were observed under a Nikon Eclipse Ti fluorescence microscope (Nikon, Tokyo, Japan). 

### 4.5. Quantification of Alkaline Phosphatase Activity 

The BioVision ALP activity colorimetric assay kit (BioVision, Inc., Milpitas, CA, USA) was used to measure the ALP activity, as previously defined, with some modifications [15]. On day 10 of osteoblastic differentiation, cells maintained in 96-well plates were rinsed with PBS and fixed with 3.7% formaldehyde in 90% ethanol for 30 s at room temperature. The fixative was replaced with 50 µL/well of p-nitrophenyl phosphate solution and cells were incubated for 30–60 min. Optical densities were then measured at 405 nm using a SpectraMax/M5 fluorescence spectrophotometer plate reader, and ALP activity was normalised to cell number.

### 4.6. Alkaline Phosphatase Staining 

On day 10 of osteoblastic differentiation, ALP staining was conducted on cells maintained in 6-well plates as previously defined [15]. Cells were fixed with 10 mM acetone/citrate buffer (pH 4.2) for 5 min at room temperature. The fixative was replaced with Naphthol/Fast Red stain for 1 h at room temperature. The stain was prepared by mixing equal amounts of 0.2 mg/mL Naphthol AS-TR phosphate substrate and 0.417 mg/mL of Fast Red stain, all reagents were purchased from Sigma, St. Louis, MO, USA. Cells were then washed with water 3 times and images were acquired under a microscope.

### 4.7. Alizarin Red S Staining of Mineralised Matrix Formation 

Alizarin red staining was conducted on day 21 of osteoblastic differentiation using the 2% Alizarin Red S Staining Kit (ScienceCell, Research Laboratories, Cat. No. 0223), as previously defined [15]. Cells were rinsed with PBS and fixed with 4% paraformaldehyde for 10 min at room temperature. Subsequently, cells were washed 3 times with distilled water and stained with the 2% Alizarin Red S stain for 10–20 min at room temperature. Finally, the cells were rinsed with water and images were acquired under a microscope.

### 4.8. RNA Extraction and cDNA Synthesis 

On day 10 of osteoblastic differentiation, total RNA was extracted from cell pellets using the total RNA Purification Kit (Norgen Biotek Corp., Thorold, ON, Canada) according the manufacturer’s instructions, as previously defined [15]. The concentrations of the isolated total RNA were determined using a NanoDrop 2000 instrument (Thermo Fisher Scientific, Waltham, MA, USA). cDNA synthesis was conducted with 500 ng of total RNA using a High Capacity cDNA Transcription Kit (Thermo Fisher Scientific Life Sciences, Waltham, MA, USA), according to the manufacturer’s instructions. 

### 4.9. Quantitative Real Time-Polymerase Chain Reaction

Quantitative real time-polymerase chain reaction (RT-PCR) was conducted on an Applied Biosystems ViiA™ 7 Real-Time PCR System (Thermo Fisher Scientific Life Sciences, Waltham, MA, USA) using fast SYBR Green. The primers used in the present study are listed in Table 1. Relative expression was calculated using the 2∆CT value method, and analysis was conducted as previously defined [48].

### 4.10. Statistical Analysis 

Statistical analysis and graphing were carried out using Microsoft Excel 2010 and the GraphPad Prism 6.0 software (GraphPad, San Diego, CA, USA), respectively. The results are presented as the mean ± SEM of at least two independent experiments. Unpaired, two-tailed Student’s *t*-test was used to assess statistical significance, and *p*-values < 0.05 were set as being statistically significant.

## 5. Conclusions

Fedratinib has been approved by the FDA for the treatment of myelofibrosis. Our data suggest the plausible use of Fedratinib to treat bone diseases characterised by increased bone formation and mineralisation such as sclerotic bone metastases, craniosynostosis, and heart valve calcification. However, the clinical efficiency of Fedratinib in these conditions requires further studies. 

## Figures and Tables

**Table 1:** List of SYBR Green primers used in present study.

Gene Name	Forward Primer	Reverse Primer
ACTB	5′AGCCATGTACGTTGCTA	5′AGTCCGCCTAGAAGCA
JAK2	CTGGAGGTCGTTCAGAGCC	CTGCTTCTGAAACCCGGC
STAT5	CTGCGAGTCTGCTACTGCTA	TGCGTTCACAAACTCAGGGA
STAT3	CAGGAGCATCCTGAAGCTGA	TGCAGGTCGTTGGTGTCA
ERK	ATCTTAAATTTGTCAGGACAAGGG	ACTGGGAAGAAGAACACCGAT
AKT	ACTCTTTCCAGACCCACGAC	ACAGGTGGAAGAACAGCTCG
ALPL	5′GGAACTCCTGACCCTTGACC3′	5′TCCTGTTCAGCTCGTACTGC3′
ON	5′GAGGAAACCGAAGAGGAGG3′	5′GGGGTGTTGTTCTCATCCAG3′
OC	GGCAGCGAGGTAGTGAAGAG	CTCACACACCTCCCTCCTG
RUNX2	5′GTAGATGGACCTCGGGAACC3′	5′GAGGCGGTCAGAGAACAAAC3′
OPN	GGTGATGTCCTCGTCTGTA	CCAAGTAAGTCCAACGAAAG
COL1A1	5′GAGTGCTGTCCCGTCTGC3′	5′TTTCTTGGTCGGTGGGTG3′
LIF	5′GCCACCCATGTCACAACAAC	5′CCCCCTGGGCTGTGTAATAG
SOCS3	5′TTCGGGACCAGCCCCC3′	5′AAACTTGCTGTGGGTGACCA3′
RRAD	5′GCGGAAACCCTAAAGTCCGA	5′GTCCGGGACCGTCCACT
NOTCH3	5′CCTGTGGCCCTCATGGTATC	5′CATGGGTTGGGGTCACAGTC
TNF	5′ACTTTGGAGTGATCGGCC3′	5′GCTTGAGGGTTTGCTACAAC3′
COMP	5′CCGACACCGCCTGCGTTCTT3′	5′AGCGCCGCGTTGGTTTCCTG3′
THBS2	5′TTGGCAAACCAGGAGCTCAG3′	5′GGTCTTGCGGTTGATGTTGC3′
IL6	CGAGCCCACCGGGAACGAAA	GGACCGAAGGCGTTGTGGAG

## Data Availability

No new data were created or analyzed in this study. Data sharing is not applicable to this article.

## Sample Availability

Not available.
